# Crystal structure and Hirshfeld surface analyses, inter­action energy calculations and energy frameworks of (*Z*)-4-benzyl-2-(4-methyl­benzylidene)-2*H*-[1,4]benzo­thia­zin-3(4*H*)-one

**DOI:** 10.1107/S2056989026004664

**Published:** 2026-05-15

**Authors:** Brahim Hni, Noureddine Hamou Ahabchane, Daouda Ballo, Tuncer Hökelek, Joel T. Mague, El Mokhtar Essassi, Nada Kheira Sebbar

**Affiliations:** a Laboratory of Heterocyclic Organic Chemistry Medicines Science Research Center Pharmacochemistry Competence Center Mohammed V University in Rabat Faculté des Sciences Av Ibn Battouta BP 1014 Rabat Morocco; b Laboratory of Constitution and Reaction of Matter (LCRM), UFR SSMT, Félix Houphouët Boigny University, 22 BP 582 Abidjan 22, Republic of Côte d’Ivoire; c Department of Physics, Hacettepe University, 06800 Beytepe, Ankara, Türkiye; d Department of Chemistry, Tulane University, New Orleans, LA 70118, USA; Vienna University of Technology, Austria

**Keywords:** crystal structure, 1,4-benzo­thia­zin-3-one, C—H⋯π(ring), weak hydrogen-bonding

## Abstract

The thia­zine ring in the title mol­ecule, C_23_H_19_NOS, exhibits a screw-boat conformation and is significantly folded along the S⋯N axis. In the extended structure, aided by C—H⋯π(ring) inter­actions, the mol­ecules pack in wave-like layers parallel to the *bc* plane. A Hirshfeld surface analysis of the crystal structure indicates that the most important contributions for the crystal packing are from H⋯H (50.3%) and H⋯C/C⋯H (35.9%) inter­actions. An evaluation of the electrostatic, dispersion and total energy frameworks in the crystal structure indicates that dispersion energy contribution dominates.

## Abstract 2

The thia­zine ring exhibits a screw-boat conformation and is significantly folded along the S⋯N axis. In the crystal, mol­ecules pack in wave-like layers parallel to the *bc* plane aided by C—H⋯π(ring) inter­actions.

## 1. Chemical context

Heterocyclic compounds containing both nitro­gen and sulfur atoms occupy a prominent position in organic chemistry due to their structural diversities and the wide range of biological activities they display (Sebbar *et al.*, 2020 ▸). Among these systems, 1,4-benzo­thia­zine derivatives represent an important class of fused heterocycles that have been extensively investigated in medicinal chemistry (Sebbar *et al.*, 2016*a* ▸,*b* ▸; Tawada *et al.*, 1990 ▸; Zia-ur-Rehman *et al.*, 2009 ▸). The inter­est in these compounds mainly arises from the diversities of their pharmacological properties, which make the 1,4-benzo­thia­zine core a valuable structural unit for the development of new therapeutic agents. Previous studies have indicated that mol­ecules containing this scaffold exhibit anti-inflammatory (Park *et al.*, 2002 ▸), anti­microbial (Rathore *et al.*, 2006 ▸), anti­pyretic (Warren *et al.*, 1987 ▸), anti­viral (Malagu *et al.*, 1998 ▸), or anti­cancer activities (Gupta *et al.*, 1986 ▸). Beyond pharmaceutical applications, 1,4-benzo­thia­zine derivatives have also gained attention as functional agents in agrochemical applications, especially as herbicides (Takemoto *et al.*, 1994 ▸), and as corrosion inhibitors for metallic materials (Ellouz *et al.*, 2016 ▸).

The sustained inter­est in the family of 1,4-benzo­thia­zines and its derivatives is largely attributed to the ease with which their mol­ecular structures can be modified, enabling the design of new compounds with enhanced physicochemical, biological or medicinal properties (Hni *et al.*, 2019 ▸). As part of our ongoing studies of N-substituted 1,4-benzo­thia­zine deriv­atives and the investigations of their potential pharmacological properties, we report herein the synthesis and crystal structure determination of (*Z*)-4-benzyl-2-(4-methyl­benzyl­idene)-2*H*-1,4-benzo­thia­zin-3(4*H*)-one, **I**[Chem scheme1]. A Hirshfeld surface analysis and evaluation of inter­molecular inter­action energies and energy frameworks complement the crystallographic study.
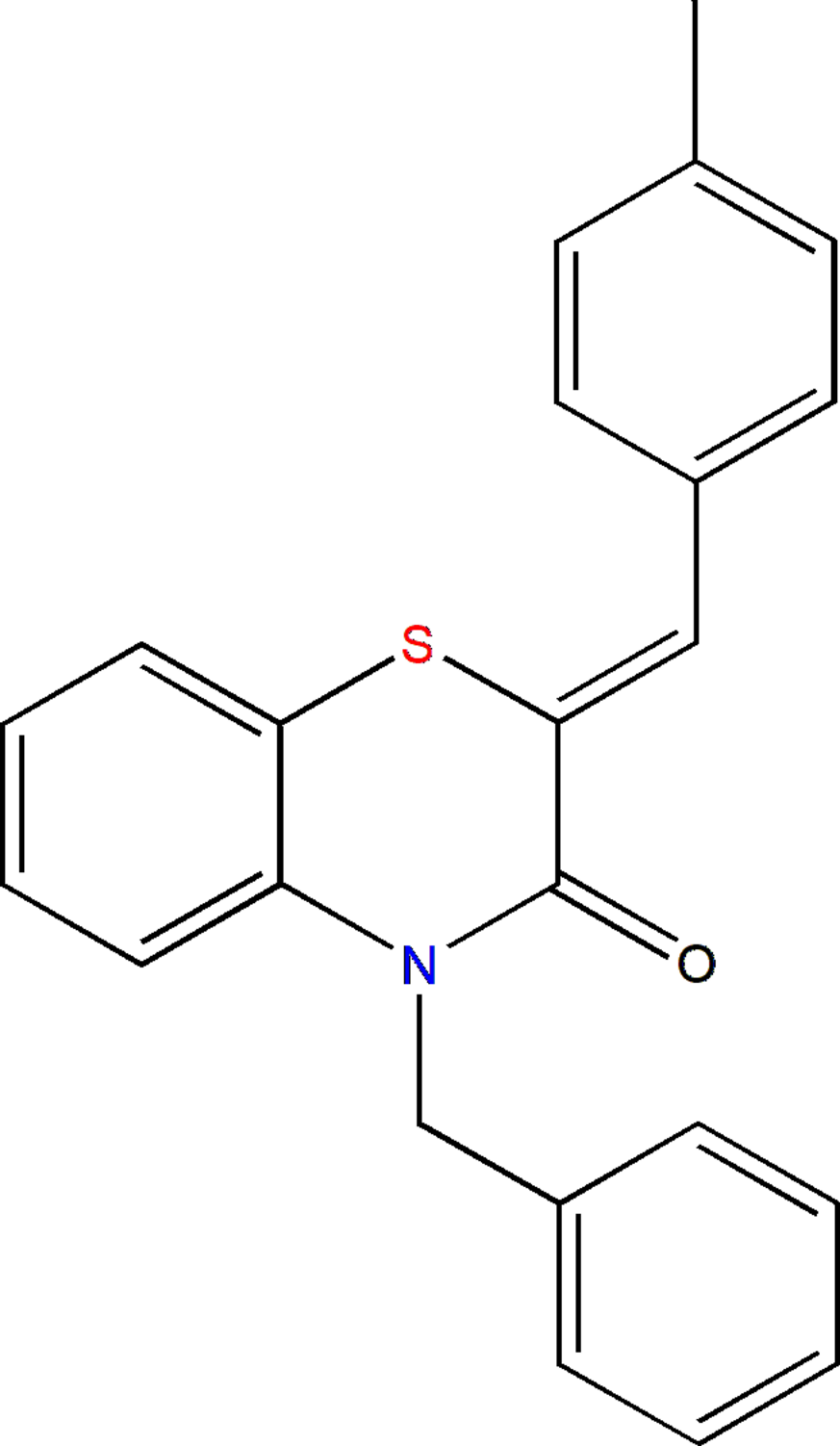


## 2. Structural commentary

**Figure 1:**
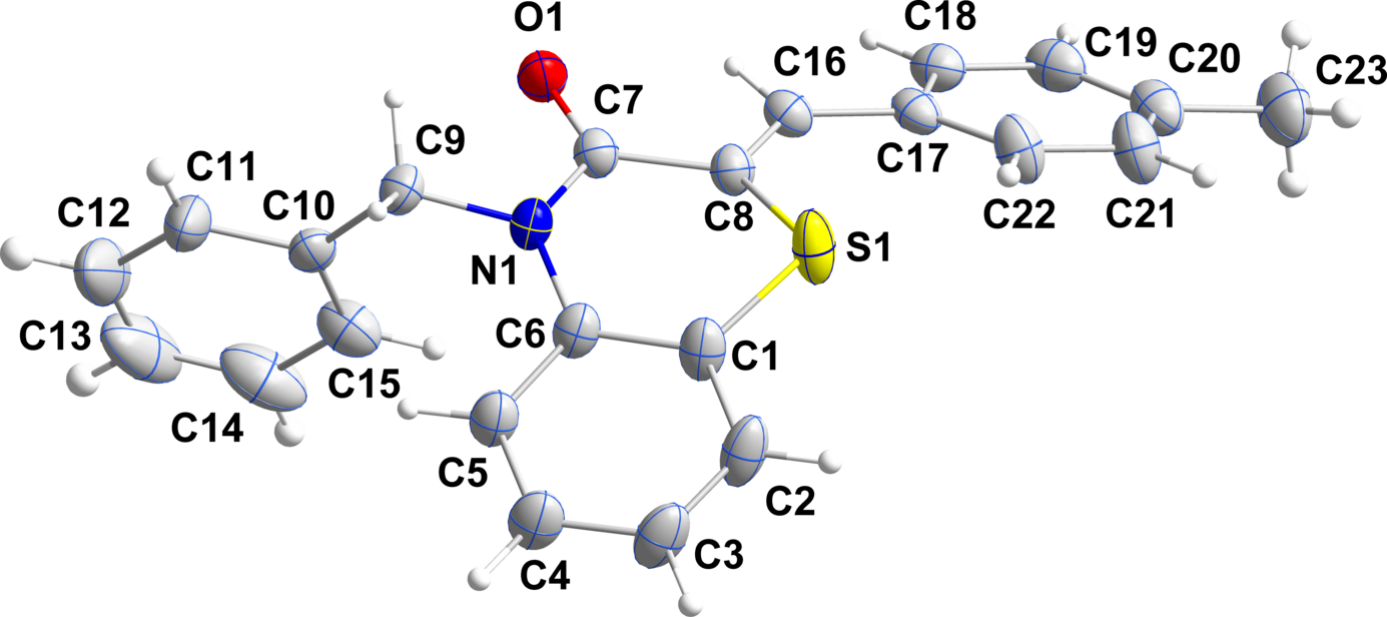
The title mol­ecule with the atom-labelling scheme and displacement ellipsoids drawn at the 50% probability level.

**Figure 2:**
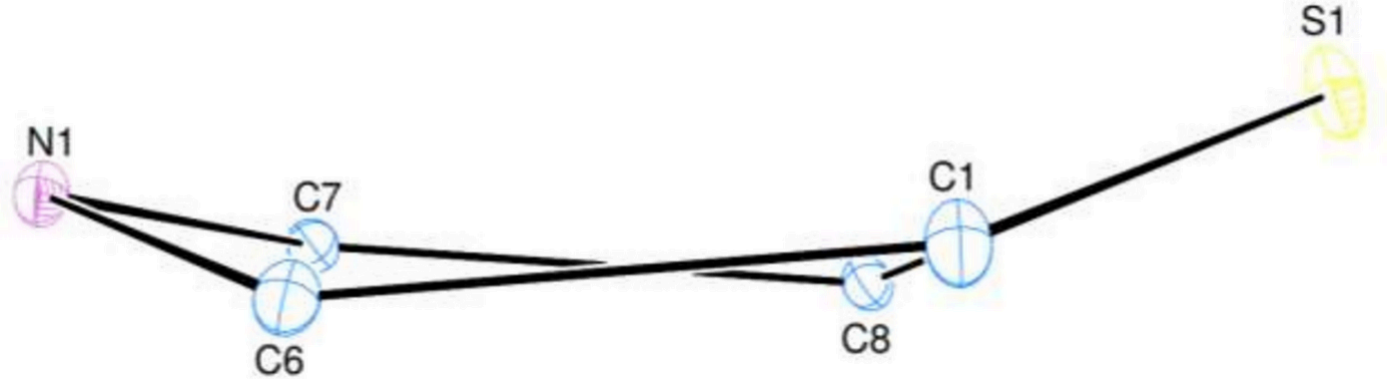
The conformation of the thia­zine ring.

In the title mol­ecule (Fig. 1 ▸), the benzo­thia­zine moiety is folded along the S1⋯N1 axis by 23.3 (1)°, which puts it in the upper third of fold angles found for these types of mol­ecules (Sebbar *et al.*, 2014 ▸). The thia­zine ring is in a screw-boat conformation (Fig. 2 ▸) with puckering parameters (Cremer & Pople, 1975 ▸) *Q*_T_ = 0.3565 (16) Å, θ = 75.4 (3)° and φ = 341.4 (3)°. The C10–C15 and C17–C22 benzene rings are inclined to the C1–C6 benzene ring by 84.22 (9) and 39.48 (8)°, respectively. This gives the mol­ecule an overall convex shape with atom H15 of the benzyl group pointing towards the concave underside (Fig. 1 ▸).

## 3. Supra­molecular features

**Figure 3:**
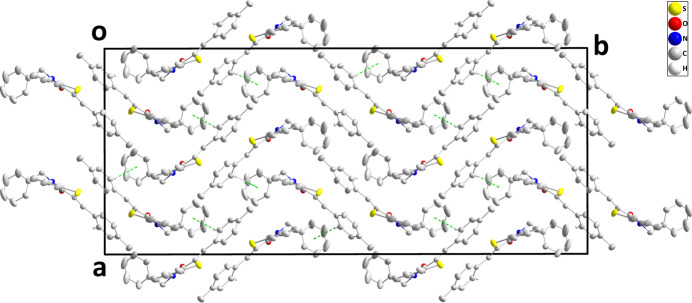
Packing of mol­ecules as viewed along the *c* axis, with C—H⋯π(ring) inter­actions depicted by dashed lines.

In the crystal, the mol­ecules pack in wave-like layers parallel to the *bc* plane with the only directed inter­actions between them being the C18—H18⋯*Cg*3 inter­action (Table 1 ▸, Fig. 3 ▸).

## 4. Hirshfeld surface analysis and energy calculations

**Figure 4:**
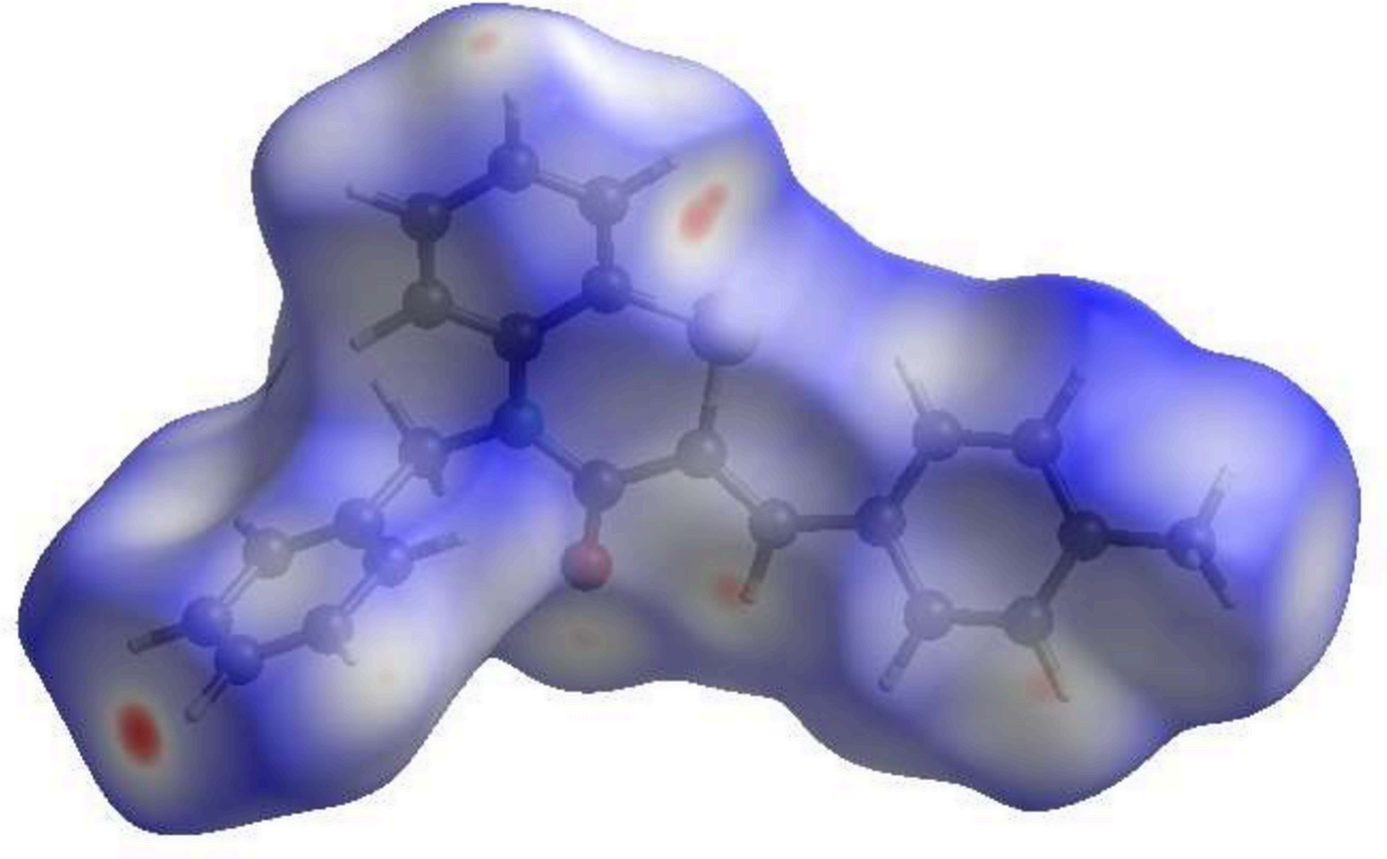
View of the HS of the title mol­ecule plotted over *d*_norm_.

**Figure 5:**
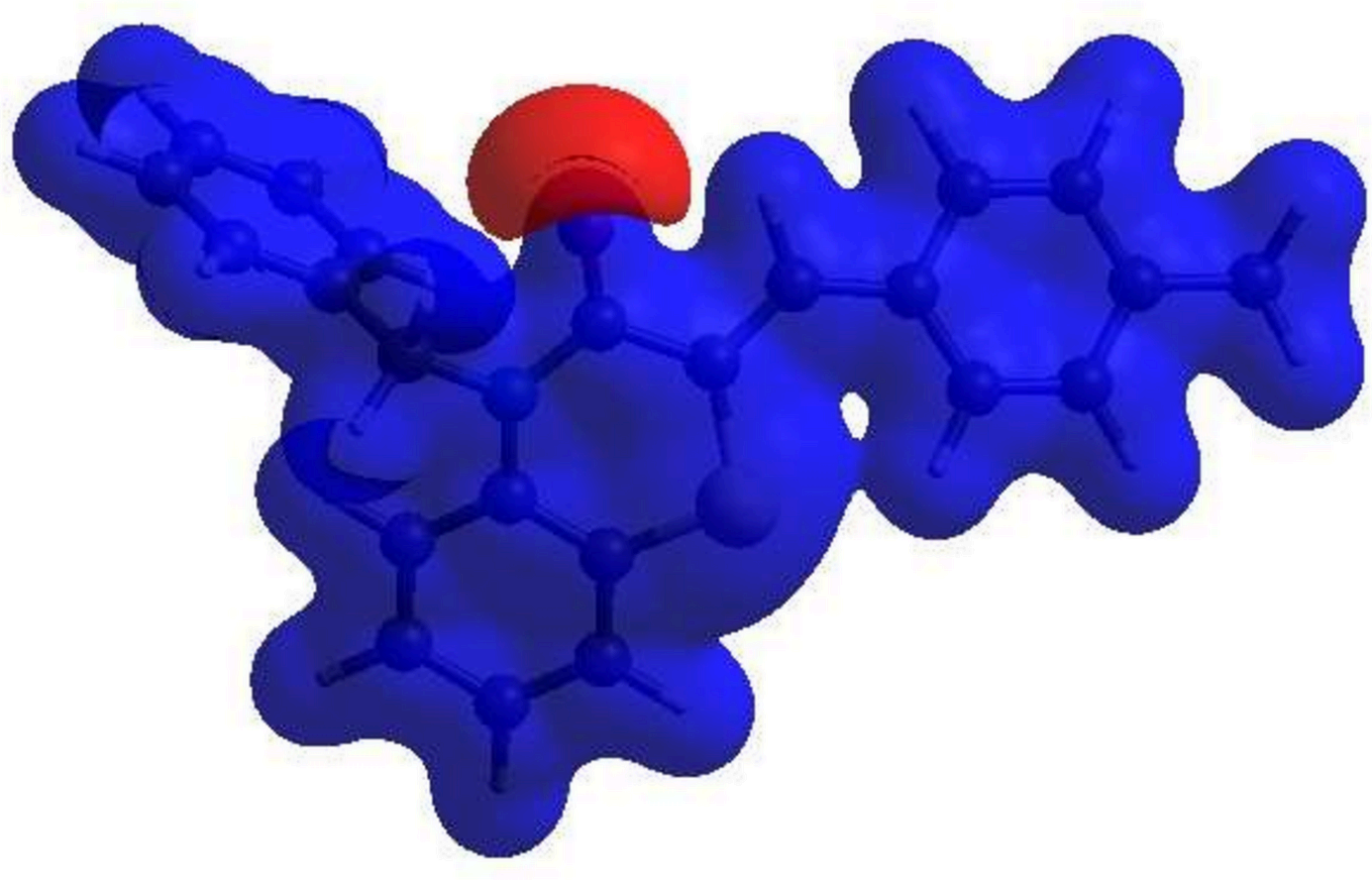
View of the HS of the title mol­ecule plotted over electrostatic potential using the STO-3 G basis set at the Hartree–Fock level of theory. Hydrogen-bonding donors and acceptors are shown as blue and red regions around the atoms, corresponding to positive and negative potentials, respectively.

**Figure 6:**
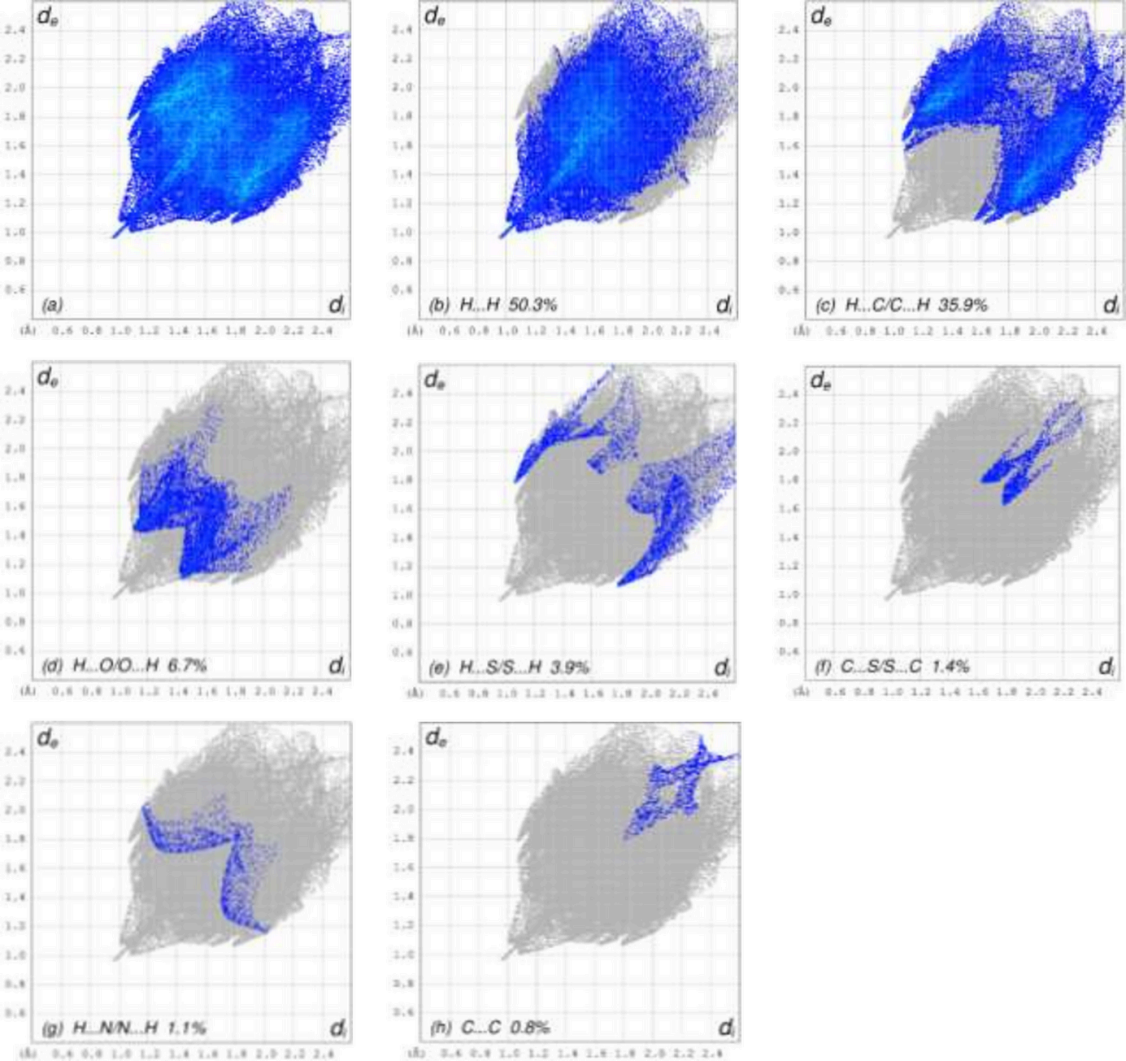
The two-dimensional fingerprint plots of the title compound, showing (*a*) all inter­actions, and delineated into (*b*) H⋯H, (*c*) H⋯C/C⋯H, (*d*) H⋯O/O⋯H, (*e*) H⋯S/S⋯H, (*f*) C⋯S/S⋯C, (*g*) H⋯N/N⋯H and (*h*) C⋯C inter­actions. The *d*_i_ and *d*_e_ values are the closest inter­nal and external distances (in Å) from given points on the Hirshfeld surface contacts.

The inter­molecular inter­actions in the crystal were qu­anti­fied by a Hirshfeld surface (HS) analysis using *CrystalExplorer* (Spackman *et al.*, 2021 ▸). Fig. 4 ▸ shows the HS mapped over *d*_norm_. The white surface indicates contacts with distances equal to the sum of van der Waals radii, and the red and blue colours indicate distances shorter (in close contact) or longer (distinct contacts) than the van der Waals radii, respectively. Hence, the red spots indicate their roles as the respective donors and/or acceptors atoms; they also appear as the blue and red regions corresponding to positive and negative potentials on the HS mapped over electrostatic potential as shown in Fig. 5 ▸. The blue and red regions indicate positive (hydrogen-bond donors) and negative (hydrogen-bond acceptors) electrostatic potentials. The overall two-dimensional fingerprint plot is shown in Fig. 6 ▸*a* and those delineated into various contact types are illustrated in Fig. 6 ▸*b–h*. According to the fingerprint plots, H⋯H and H⋯C/C⋯H contacts make the most significant contributions to the HS, at 50.3% and 35.9%, respectively.

**Figure 7:**
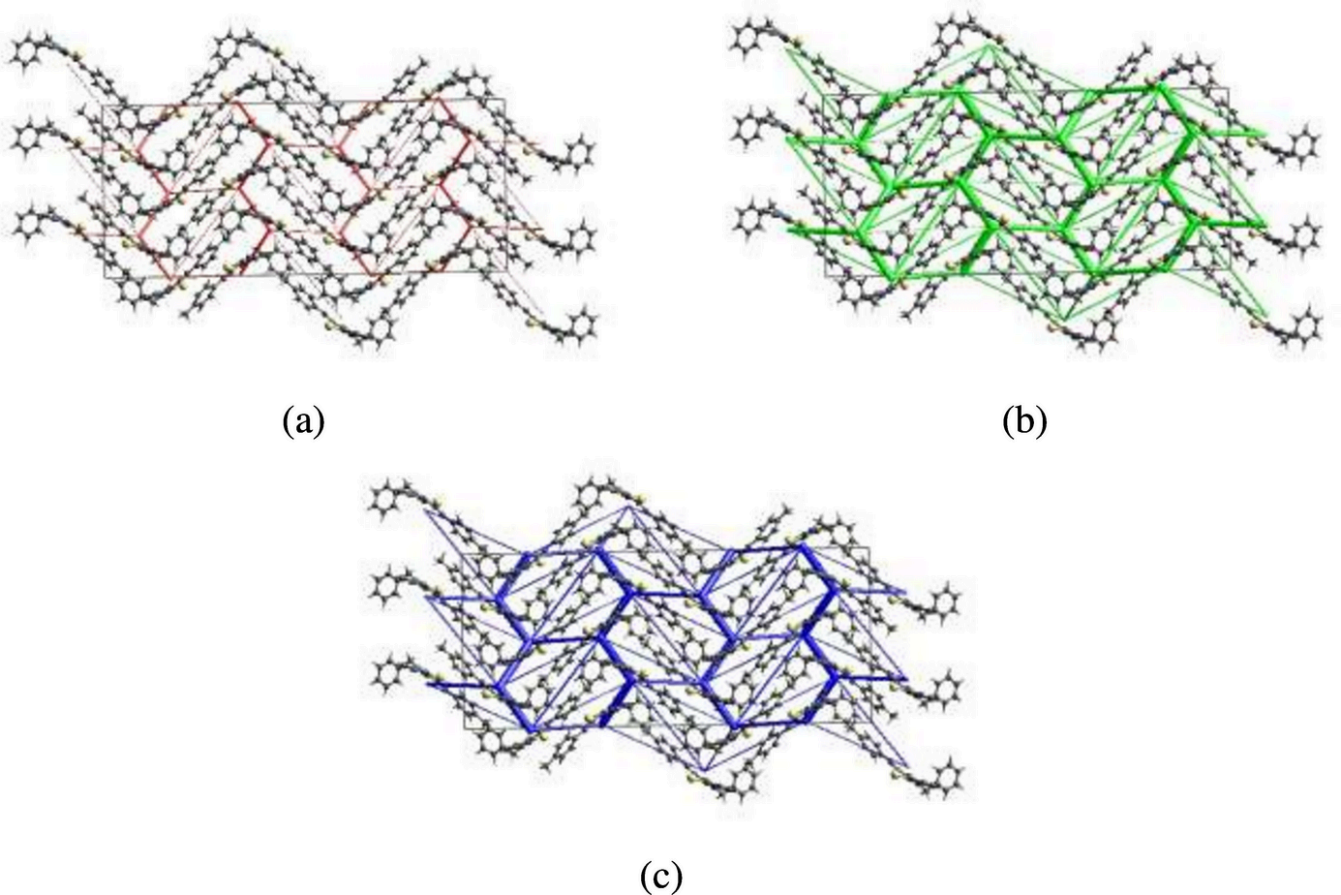
The energy frameworks for a cluster of mol­ecules of the title compound viewed down the *c* axis showing the (*a*) electrostatic energy, (*b*) dispersion energy and (*c*) total energy diagrams. The cylindrical radius is proportional to the relative strength of the corresponding energies and they were adjusted to the same scale factor of 80 with cut-off value of 5 kJ mol^−1^ within the unit cell.

The inter­molecular inter­action energies were calculated using the CE–B3LYP/6–31G(d,p) energy model available in *CrystalExplorer*, where a cluster of mol­ecules is generated by applying crystallographic symmetry operations with respect to a selected central mol­ecule within a radius of 3.8 Å by default. The maximum inter­action energy occurring at 6.14 Å with an *E*_total_ value of −56.1 kJ mol^−1^ is dominated by the dispersion component of *E*_dis_ = −67.4 kJ mol^−1^ that is significantly larger than the electrostatic component of *E*_ele_ = −20.4 kJ mol^−1^. Energy frameworks combine the calculation of inter­molecular inter­action energies with a graphical representation of their magnitudes, in which they were constructed for *E*_ele_ (red cylinders), *E*_dis_ (green cylinders) and *E*_tot_ (blue cylinders), as shown in Fig. 7 ▸*a*, *b* and *c*, respectively. The evaluation of these frameworks indicates that the stabilization is dominated *via* the dispersion energy contributions.

## 5. Database survey

**Figure 8:**
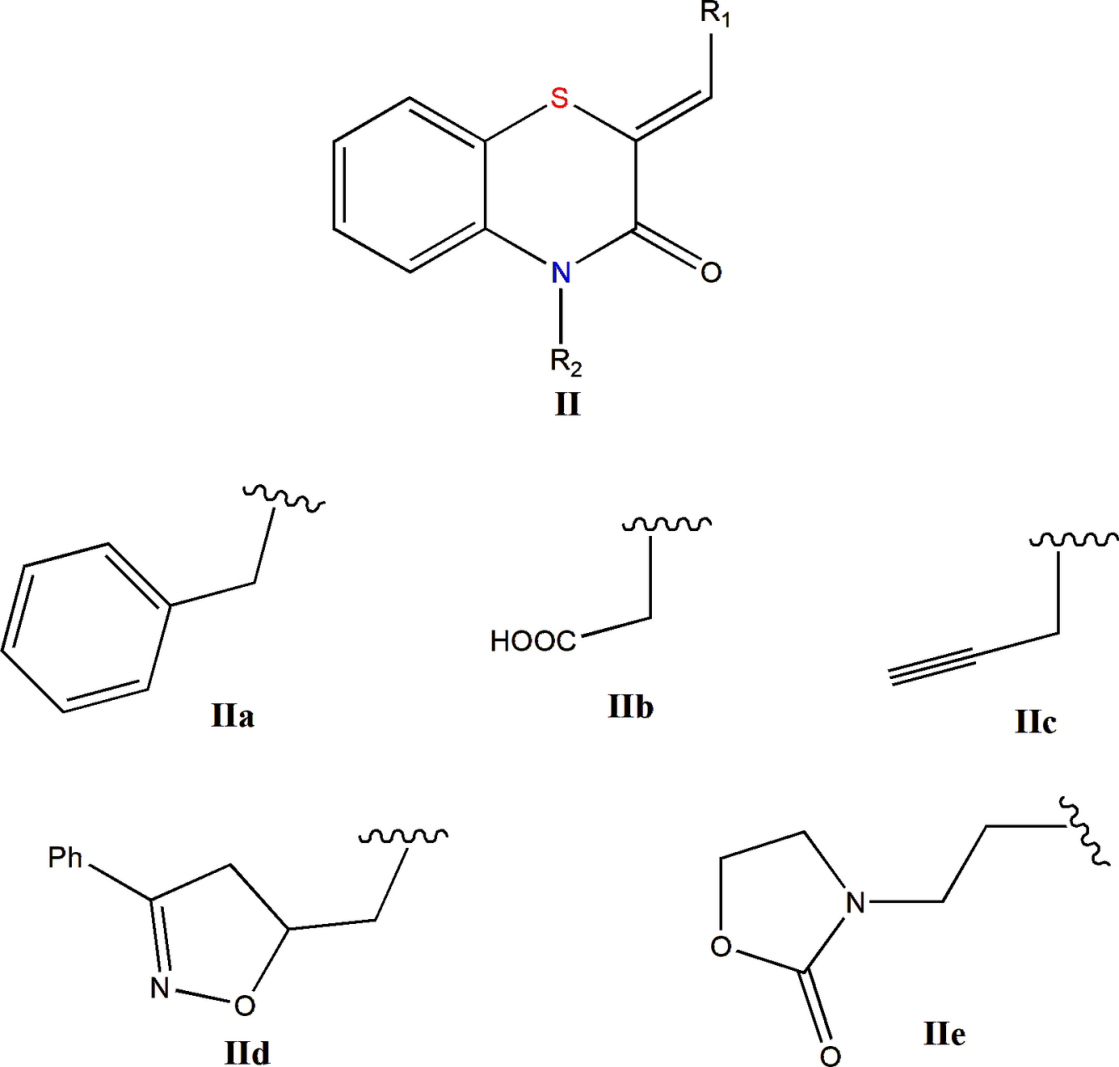
Schematic representation of candidates for the search in the CSD.

A survey of the Cambridge Structural Database (CSD; Groom *et al.*, 2016 ▸; update of March 2026) for structures incorporating fragment **II** (*R*1 = Ph, *R*2 = C; Fig. 8 ▸) identified 14 related entries. Among these, compounds **IIa** correspond to derivatives bearing *R*1 = 4-ClC_6_H_4_ or 2,4-ClC_6_H_4_ and *R*2 = CH_2_Ph_2_ (Sebbar *et al.*, 2019 ▸). Compound **IIb** has been reported with *R*1 = 4-ClC_6_H_4_ and *R*2 = CH_2_COOH (Sebbar *et al.*, 2016*a* ▸), whereas compounds **IIc** include examples with *R*1 = Ph, 4-FC_6_H_4_, or 2-ClC_6_H_4_ and *R*2 = CH_2_C≡CH (Hni *et al.*, 2019 ▸). Additional related structures correspond to types **IId** and **IIe** (Sebbar *et al.*, 2016*b* ▸). In every case, the benzyl­idene C=CHC_6_H_5_ double bond leads to a *Z* configuration. Furthermore, most of these structures display a markedly non-planar heterocyclic ring. The dihedral angle between the plane formed by the benzene ring together with the nitro­gen and sulfur atoms, and the plane defined by the nitro­gen and sulfur atoms and the two inter­vening carbon atoms, varies from approximately 29° in **IIc** to 36° in **IId**.

## 6. Synthesis and crystallization

To a solution of (Z)-2-(4-methyl­benzyl­idene)-2*H*-1,4-benzo­thia­zin-3(4*H*)-one (3.21 mmol), benzyl chloride (6.52 mmol) and potassium carbonate (6.51 mmol) in di­methyl­formamide (DMF; 20ml), a catalytic amount of tetra-*n*-butyl ammonium bromide (0.33 mmol) was added. The mixture was then stirred for 24 h. The solid material was removed by filtration and the solvent evaporated under vacuum. The solid product was purified by recrystallization from ethanol to afford colourless crystals in 86% yield.

## 7. Refinement

Crystal data, data collection and structure refinement details are summarized in Table 2 ▸. C-bound H atoms were positioned geometrically (C—H = 0.95–0.99 Å) and were included as riding contributions with isotropic displacement parameters 1.2–1.5 times those of the attached atoms. The phenyl group (C10–C15) of the benzyl moiety suffers from minor disorder as evidenced by the elongated displacement ellipsoids for most of the atoms. Attempts to model the disorder with two rigid groups led to an unstable refinement and were not pursued.

## Supplementary Material

Crystal structure: contains datablock(s) global, I. DOI: 10.1107/S2056989026004664/wm5798sup1.cif

Structure factors: contains datablock(s) I. DOI: 10.1107/S2056989026004664/wm5798Isup2.hkl

Supporting information file. DOI: 10.1107/S2056989026004664/wm5798Isup3.cdx

Supporting information file. DOI: 10.1107/S2056989026004664/wm5798Isup4.cml

CCDC reference: 2551702

Additional supporting information:  crystallographic information; 3D view; checkCIF report

## Figures and Tables

**Table 1:** Hydrogen-bond geometry (Å, °) *Cg*3 is the centroid of the C10–C15 benzene ring.

*D*—H⋯*A*	*D*—H	H⋯*A*	*D*⋯*A*	*D*—H⋯*A*
C18—H18⋯*Cg*3^i^	0.95	3.00	3.898 (2)	158

Symmetry code: (i) 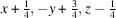.

**Table 2:** Experimental details

Crystal data	
Chemical formula	C_23_H_19_NOS
*M*_r_	357.45
Crystal system, space group	Orthorhombic, *Fdd*2
Temperature (K)	150
*a*, *b*, *c* (Å)	18.7286 (11), 43.903 (2), 8.9154 (5)
*V* (Å^3^)	7330.5 (7)
*Z*	16
Radiation type	Mo *K*α
μ (mm^−1^)	0.19
Crystal size (mm)	0.36 × 0.32 × 0.27

Data collection	
Diffractometer	Bruker SMART *APEX* CCD
Absorption correction	Multi-scan (*SADABS*; Krause *et al.*, 2015 ▸)
*T*_min_, *T*_max_	0.85, 0.95
No. of measured, independent and observed [*I* > 2σ(*I*)] reflections	34831, 4941, 4700
*R*_int_	0.028
(sin θ/λ)_max_ (Å^−1^)	0.688

Refinement	
*R*[*F*^2^ > 2σ(*F*^2^)], *wR*(*F*^2^), *S*	0.037, 0.093, 1.06
No. of reflections	4941
No. of parameters	236
No. of restraints	31
H-atom treatment	H-atom parameters constrained
Δρ_max_, Δρ_min_ (e Å^−3^)	0.32, −0.22
Absolute structure	Flack *x* determined using 2075 quotients [(*I*^+^)−(*I*^−^)]/[(*I*^+^)+(*I*^−^)] (Parsons *et al.*, 2013 ▸)
Absolute structure parameter	0.010 (13)

Computer programs: *APEX3* and *SAINT* (Bruker, 2016 ▸), *SHELXT* (Sheldrick, 2015*a* ▸), *SHELXL* (Sheldrick, 2015*b* ▸), *DIAMOND* (Brandenburg & Putz, 2012 ▸) and *publCIF* (Westrip, 2010 ▸).

## supplementary crystallographic information

### (Z)-4-Benzyl-2-(4-methylbenzylidene)-2H-benzo[b][1,4]thiazin-3(4H)-one Crystal data

**Table:**

C_23_H_19_NOS	*D*_x_ = 1.296 Mg m^−^^3^
*M**_r_* = 357.45	Mo *K*α radiation, λ = 0.71073 Å
Orthorhombic, *F**d**d*2	Cell parameters from 9879 reflections
*a* = 18.7286 (11) Å	θ = 2.4–29.2°
*b* = 43.903 (2) Å	µ = 0.19 mm^−^^1^
*c* = 8.9154 (5) Å	*T* = 150 K
*V* = 7330.5 (7) Å^3^	Block, colourless
*Z* = 16	0.36 × 0.32 × 0.27 mm
*F*(000) = 3008	

### (Z)-4-Benzyl-2-(4-methylbenzylidene)-2H-benzo[b][1,4]thiazin-3(4H)-one Data collection

**Table:**

Bruker SMART APEX CCD diffractometer	4941 independent reflections
Radiation source: fine-focus sealed tube	4700 reflections with *I* > 2σ(*I*)
Graphite monochromator	*R*_int_ = 0.028
Detector resolution: 8.3333 pixels mm^-1^	θ_max_ = 29.3°, θ_min_ = 1.9°
φ and ω scans	*h* = −25→25
Absorption correction: multi-scan (*SADABS*; Krause *et al.*, 2015)	*k* = −60→60
*T*_min_ = 0.85, *T*_max_ = 0.95	*l* = −12→12
34831 measured reflections	

### (Z)-4-Benzyl-2-(4-methylbenzylidene)-2H-benzo[b][1,4]thiazin-3(4H)-one Refinement

**Table:**

Refinement on *F*^2^	Secondary atom site location: difference Fourier map
Least-squares matrix: full	Hydrogen site location: inferred from neighbouring sites
*R*[*F*^2^ > 2σ(*F*^2^)] = 0.037	H-atom parameters constrained
*wR*(*F*^2^) = 0.093	*w* = 1/[σ^2^(*F*_o_^2^) + (0.0567*P*)^2^ + 3.7343*P*] where *P* = (*F*_o_^2^ + 2*F*_c_^2^)/3
*S* = 1.06	(Δ/σ)_max_ = 0.001
4941 reflections	Δρ_max_ = 0.32 e Å^−^^3^
236 parameters	Δρ_min_ = −0.22 e Å^−^^3^
31 restraints	Absolute structure: Flack *x* determined using 2075 quotients [(*I*^+^)-(*I*^-^)]/[(*I*^+^)+(*I*^-^)] (Parsons *et al.*, 2013)
Primary atom site location: dual	Absolute structure parameter: 0.010 (13)

### (Z)-4-Benzyl-2-(4-methylbenzylidene)-2H-benzo[b][1,4]thiazin-3(4H)-one Special details

**Table:**

Experimental. The diffraction data were obtained from 3 sets of 400 frames, each of width 0.5° in ω, colllected at φ = 0.00, 90.00 and 180.00° and 2 sets of 800 frames, each of width 0.45° in φ, collected at ω = –30.00 and 210.00°. The scan time was 15 sec/frame.
Geometry. All esds (except the esd in the dihedral angle between two l.s. planes) are estimated using the full covariance matrix. The cell esds are taken into account individually in the estimation of esds in distances, angles and torsion angles; correlations between esds in cell parameters are only used when they are defined by crystal symmetry. An approximate (isotropic) treatment of cell esds is used for estimating esds involving l.s. planes.
Refinement. Refinement of F^2^ against ALL reflections. The weighted R-factor wR and goodness of fit S are based on F^2^, conventional R-factors R are based on F, with F set to zero for negative F^2^. The threshold expression of F^2^ > 2sigma(F^2^) is used only for calculating R-factors(gt) etc. and is not relevant to the choice of reflections for refinement. R-factors based on F^2^ are statistically about twice as large as those based on F, and R- factors based on ALL data will be even larger. H-atoms attached to carbon were placed in calculated positions (C—H = 0.95 - 0.99 Å). All were included as riding contributions with isotropic displacement parameters 1.2 - 1.5 times those of the attached atoms. The phenyl group C10···C15 suffers from minor disorder as evidenced by the elongated displacement ellipsoids for most of the atoms. Attempts to model the disorder with two rigid hexagon sites led to an unstable refinement and so were not pursued.

### (Z)-4-Benzyl-2-(4-methylbenzylidene)-2H-benzo[b][1,4]thiazin-3(4H)-one Fractional atomic coordinates and isotropic or equivalent isotropic displacement parameters (Å^2^)

**Table:**

	*x*	*y*	*z*	*U*_iso_*/*U*_eq_	
S1	0.19870 (3)	0.44442 (2)	0.60481 (6)	0.04254 (16)	
O1	0.18669 (9)	0.41154 (3)	0.19620 (17)	0.0356 (3)	
N1	0.14611 (9)	0.39080 (4)	0.41107 (19)	0.0284 (3)	
C1	0.17135 (11)	0.40843 (5)	0.6666 (2)	0.0335 (4)	
C2	0.17002 (12)	0.40339 (6)	0.8212 (3)	0.0416 (5)	
H2	0.183487	0.419267	0.887892	0.050*	
C3	0.14930 (13)	0.37555 (6)	0.8777 (3)	0.0430 (5)	
H3	0.148292	0.372284	0.983053	0.052*	
C4	0.12992 (13)	0.35231 (5)	0.7805 (3)	0.0389 (5)	
H4	0.116288	0.333005	0.819175	0.047*	
C5	0.13045 (11)	0.35728 (5)	0.6267 (2)	0.0333 (4)	
H5	0.117455	0.341240	0.560583	0.040*	
C6	0.14986 (10)	0.38557 (4)	0.5680 (2)	0.0291 (4)	
C7	0.18423 (10)	0.41240 (4)	0.3334 (2)	0.0280 (4)	
C8	0.22171 (10)	0.43711 (4)	0.4178 (2)	0.0282 (4)	
C9	0.10298 (10)	0.37004 (4)	0.3195 (2)	0.0284 (4)	
H9A	0.085046	0.381357	0.231188	0.034*	
H9B	0.061013	0.363559	0.379031	0.034*	
C10	0.14167 (11)	0.34193 (4)	0.2652 (2)	0.0293 (4)	
C11	0.10357 (19)	0.32103 (6)	0.1796 (3)	0.0575 (8)	
H11	0.054201	0.324269	0.160976	0.069*	
C12	0.1371 (3)	0.29553 (7)	0.1213 (4)	0.0831 (12)	
H12	0.110860	0.281341	0.062798	0.100*	
C13	0.2088 (3)	0.29079 (7)	0.1484 (4)	0.0809 (12)	
H13	0.232125	0.273554	0.106422	0.097*	
C14	0.24653 (18)	0.31078 (7)	0.2353 (4)	0.0647 (9)	
H14	0.295524	0.307112	0.256186	0.078*	
C15	0.21316 (12)	0.33643 (5)	0.2931 (3)	0.0413 (5)	
H15	0.239716	0.350386	0.352412	0.050*	
C16	0.26745 (10)	0.45466 (4)	0.3393 (2)	0.0290 (4)	
H16	0.270036	0.449685	0.235720	0.035*	
C17	0.31382 (10)	0.47992 (4)	0.3834 (2)	0.0300 (4)	
C18	0.36728 (11)	0.48828 (5)	0.2807 (2)	0.0336 (4)	
H18	0.370584	0.477985	0.187258	0.040*	
C19	0.41524 (11)	0.51136 (5)	0.3141 (3)	0.0378 (5)	
H19	0.450342	0.516830	0.241997	0.045*	
C20	0.41322 (11)	0.52667 (5)	0.4501 (3)	0.0383 (5)	
C21	0.35937 (13)	0.51898 (5)	0.5500 (3)	0.0443 (5)	
H21	0.356185	0.529493	0.642919	0.053*	
C22	0.30992 (12)	0.49626 (5)	0.5176 (3)	0.0403 (5)	
H22	0.273081	0.491817	0.587471	0.048*	
C23	0.46743 (14)	0.55090 (6)	0.4877 (4)	0.0526 (6)	
H23A	0.477091	0.563235	0.398377	0.079*	
H23B	0.511753	0.541230	0.521572	0.079*	
H23C	0.448664	0.563967	0.567616	0.079*	

### (Z)-4-Benzyl-2-(4-methylbenzylidene)-2H-benzo[b][1,4]thiazin-3(4H)-one Atomic displacement parameters (Å^2^)

**Table:**

	*U*^11^	*U*^22^	*U*^33^	*U*^12^	*U*^13^	*U*^23^
S1	0.0539 (3)	0.0364 (2)	0.0374 (3)	−0.0173 (2)	0.0175 (2)	−0.0148 (2)
O1	0.0468 (8)	0.0324 (7)	0.0275 (7)	−0.0058 (6)	−0.0001 (6)	0.0008 (5)
N1	0.0282 (7)	0.0302 (7)	0.0266 (8)	−0.0048 (6)	0.0010 (6)	−0.0049 (6)
C1	0.0332 (10)	0.0375 (9)	0.0298 (10)	−0.0110 (8)	0.0090 (8)	−0.0076 (8)
C2	0.0418 (11)	0.0550 (13)	0.0281 (10)	−0.0174 (10)	0.0069 (9)	−0.0131 (9)
C3	0.0455 (12)	0.0602 (13)	0.0231 (9)	−0.0143 (10)	0.0049 (9)	−0.0047 (9)
C4	0.0421 (11)	0.0454 (11)	0.0294 (10)	−0.0107 (9)	0.0061 (8)	0.0010 (8)
C5	0.0350 (9)	0.0363 (9)	0.0285 (10)	−0.0076 (8)	0.0032 (7)	−0.0028 (7)
C6	0.0263 (8)	0.0350 (9)	0.0260 (9)	−0.0052 (7)	0.0044 (7)	−0.0044 (7)
C7	0.0286 (8)	0.0257 (8)	0.0296 (10)	0.0006 (6)	0.0009 (7)	−0.0022 (7)
C8	0.0292 (8)	0.0267 (7)	0.0288 (8)	0.0005 (6)	0.0022 (7)	−0.0040 (7)
C9	0.0250 (8)	0.0317 (8)	0.0284 (9)	−0.0012 (6)	−0.0019 (7)	−0.0038 (7)
C10	0.0376 (10)	0.0293 (8)	0.0211 (8)	−0.0001 (7)	−0.0003 (7)	0.0009 (6)
C11	0.0805 (19)	0.0377 (11)	0.0542 (16)	0.0039 (12)	−0.0337 (15)	−0.0116 (11)
C12	0.152 (4)	0.0405 (13)	0.0570 (18)	0.0161 (18)	−0.034 (2)	−0.0204 (13)
C13	0.137 (3)	0.0443 (14)	0.0610 (19)	0.0365 (19)	0.017 (2)	−0.0041 (13)
C14	0.0648 (17)	0.0496 (14)	0.080 (2)	0.0246 (13)	0.0290 (16)	0.0164 (14)
C15	0.0356 (10)	0.0374 (10)	0.0510 (13)	0.0040 (8)	0.0091 (9)	0.0057 (9)
C16	0.0281 (8)	0.0259 (8)	0.0330 (10)	0.0021 (6)	0.0023 (7)	0.0016 (7)
C17	0.0273 (8)	0.0240 (7)	0.0387 (11)	0.0039 (6)	−0.0003 (7)	0.0042 (7)
C18	0.0311 (9)	0.0341 (9)	0.0355 (11)	0.0005 (7)	−0.0006 (8)	0.0060 (8)
C19	0.0273 (9)	0.0387 (10)	0.0474 (12)	−0.0022 (8)	0.0013 (9)	0.0108 (9)
C20	0.0292 (9)	0.0296 (9)	0.0562 (13)	−0.0002 (7)	−0.0014 (9)	0.0030 (9)
C21	0.0457 (12)	0.0315 (10)	0.0556 (14)	−0.0057 (9)	0.0097 (10)	−0.0115 (9)
C22	0.0400 (11)	0.0310 (9)	0.0498 (13)	−0.0055 (8)	0.0154 (10)	−0.0071 (9)
C23	0.0397 (12)	0.0440 (12)	0.0742 (18)	−0.0118 (10)	−0.0014 (12)	−0.0043 (12)

### (Z)-4-Benzyl-2-(4-methylbenzylidene)-2H-benzo[b][1,4]thiazin-3(4H)-one Geometric parameters (Å, º)

**Table:**

S1—C1	1.750 (2)	C11—H11	0.9500
S1—C8	1.751 (2)	C12—C13	1.380 (7)
O1—C7	1.225 (3)	C12—H12	0.9500
N1—C7	1.375 (2)	C13—C14	1.367 (6)
N1—C6	1.419 (3)	C13—H13	0.9500
N1—C9	1.466 (2)	C14—C15	1.387 (3)
C1—C6	1.393 (3)	C14—H14	0.9500
C1—C2	1.397 (3)	C15—H15	0.9500
C2—C3	1.378 (3)	C16—C17	1.463 (3)
C2—H2	0.9500	C16—H16	0.9500
C3—C4	1.387 (3)	C17—C22	1.396 (3)
C3—H3	0.9500	C17—C18	1.406 (3)
C4—C5	1.389 (3)	C18—C19	1.386 (3)
C4—H4	0.9500	C18—H18	0.9500
C5—C6	1.396 (3)	C19—C20	1.387 (3)
C5—H5	0.9500	C19—H19	0.9500
C7—C8	1.495 (3)	C20—C21	1.387 (3)
C8—C16	1.348 (3)	C20—C23	1.508 (3)
C9—C10	1.511 (3)	C21—C22	1.392 (3)
C9—H9A	0.9900	C21—H21	0.9500
C9—H9B	0.9900	C22—H22	0.9500
C10—C15	1.383 (3)	C23—H23A	0.9800
C10—C11	1.390 (3)	C23—H23B	0.9800
C11—C12	1.385 (4)	C23—H23C	0.9800
			
C1—S1—C8	101.89 (9)	C13—C12—C11	119.9 (3)
C7—N1—C6	125.62 (16)	C13—C12—H12	120.1
C7—N1—C9	115.77 (15)	C11—C12—H12	120.1
C6—N1—C9	118.39 (15)	C14—C13—C12	120.3 (3)
C6—C1—C2	120.24 (19)	C14—C13—H13	119.9
C6—C1—S1	122.43 (16)	C12—C13—H13	119.9
C2—C1—S1	117.31 (16)	C13—C14—C15	119.9 (3)
C3—C2—C1	120.4 (2)	C13—C14—H14	120.0
C3—C2—H2	119.8	C15—C14—H14	120.0
C1—C2—H2	119.8	C10—C15—C14	120.7 (3)
C2—C3—C4	119.9 (2)	C10—C15—H15	119.6
C2—C3—H3	120.1	C14—C15—H15	119.6
C4—C3—H3	120.1	C8—C16—C17	132.1 (2)
C3—C4—C5	120.0 (2)	C8—C16—H16	113.9
C3—C4—H4	120.0	C17—C16—H16	113.9
C5—C4—H4	120.0	C22—C17—C18	117.45 (18)
C4—C5—C6	120.78 (19)	C22—C17—C16	126.09 (18)
C4—C5—H5	119.6	C18—C17—C16	116.46 (19)
C6—C5—H5	119.6	C19—C18—C17	120.8 (2)
C1—C6—C5	118.66 (18)	C19—C18—H18	119.6
C1—C6—N1	121.31 (18)	C17—C18—H18	119.6
C5—C6—N1	120.02 (17)	C18—C19—C20	121.6 (2)
O1—C7—N1	120.08 (17)	C18—C19—H19	119.2
O1—C7—C8	120.52 (18)	C20—C19—H19	119.2
N1—C7—C8	119.39 (17)	C19—C20—C21	117.6 (2)
C16—C8—C7	116.82 (18)	C19—C20—C23	121.2 (2)
C16—C8—S1	123.09 (15)	C21—C20—C23	121.2 (2)
C7—C8—S1	119.77 (14)	C20—C21—C22	121.6 (2)
N1—C9—C10	114.96 (15)	C20—C21—H21	119.2
N1—C9—H9A	108.5	C22—C21—H21	119.2
C10—C9—H9A	108.5	C21—C22—C17	120.8 (2)
N1—C9—H9B	108.5	C21—C22—H22	119.6
C10—C9—H9B	108.5	C17—C22—H22	119.6
H9A—C9—H9B	107.5	C20—C23—H23A	109.5
C15—C10—C11	118.7 (2)	C20—C23—H23B	109.5
C15—C10—C9	123.32 (18)	H23A—C23—H23B	109.5
C11—C10—C9	117.9 (2)	C20—C23—H23C	109.5
C12—C11—C10	120.4 (3)	H23A—C23—H23C	109.5
C12—C11—H11	119.8	H23B—C23—H23C	109.5
C10—C11—H11	119.8		
			
C8—S1—C1—C6	−20.9 (2)	C7—N1—C9—C10	87.9 (2)
C8—S1—C1—C2	160.67 (18)	C6—N1—C9—C10	−87.1 (2)
C6—C1—C2—C3	1.7 (4)	N1—C9—C10—C15	−2.7 (3)
S1—C1—C2—C3	−179.75 (19)	N1—C9—C10—C11	179.1 (2)
C1—C2—C3—C4	0.3 (4)	C15—C10—C11—C12	−1.2 (4)
C2—C3—C4—C5	−0.9 (4)	C9—C10—C11—C12	177.1 (3)
C3—C4—C5—C6	−0.4 (4)	C10—C11—C12—C13	0.1 (6)
C2—C1—C6—C5	−3.0 (3)	C11—C12—C13—C14	1.5 (6)
S1—C1—C6—C5	178.52 (16)	C12—C13—C14—C15	−1.9 (5)
C2—C1—C6—N1	175.7 (2)	C11—C10—C15—C14	0.8 (4)
S1—C1—C6—N1	−2.8 (3)	C9—C10—C15—C14	−177.5 (2)
C4—C5—C6—C1	2.4 (3)	C13—C14—C15—C10	0.8 (4)
C4—C5—C6—N1	−176.3 (2)	C7—C8—C16—C17	−178.35 (18)
C7—N1—C6—C1	24.5 (3)	S1—C8—C16—C17	8.1 (3)
C9—N1—C6—C1	−161.06 (18)	C8—C16—C17—C22	−14.8 (3)
C7—N1—C6—C5	−156.78 (18)	C8—C16—C17—C18	165.0 (2)
C9—N1—C6—C5	17.6 (3)	C22—C17—C18—C19	1.7 (3)
C6—N1—C7—O1	167.09 (18)	C16—C17—C18—C19	−178.12 (18)
C9—N1—C7—O1	−7.5 (3)	C17—C18—C19—C20	1.1 (3)
C6—N1—C7—C8	−13.7 (3)	C18—C19—C20—C21	−2.7 (3)
C9—N1—C7—C8	171.75 (16)	C18—C19—C20—C23	177.6 (2)
O1—C7—C8—C16	−11.2 (3)	C19—C20—C21—C22	1.5 (4)
N1—C7—C8—C16	169.57 (17)	C23—C20—C21—C22	−178.8 (2)
O1—C7—C8—S1	162.57 (16)	C20—C21—C22—C17	1.3 (4)
N1—C7—C8—S1	−16.6 (2)	C18—C17—C22—C21	−2.8 (3)
C1—S1—C8—C16	−156.70 (17)	C16—C17—C22—C21	176.9 (2)
C1—S1—C8—C7	29.91 (18)		

### (Z)-4-Benzyl-2-(4-methylbenzylidene)-2H-benzo[b][1,4]thiazin-3(4H)-one Hydrogen-bond geometry (Å, º)

*Cg*3 is the centroid of the C10–C15 benzene ring.

**Table:**

*D*—H···*A*	*D*—H	H···*A*	*D*···*A*	*D*—H···*A*
C18—H18···*Cg*3^i^	0.95	3.00	3.898 (2)	158

Symmetry code: (i) *x*+1/4, −*y*+3/4, *z*−1/4.
